# ‘You've Given Me My Voice’—Enhancing Engagement in Exercise Oncology Research: A Patient and Public Involvement Approach to Co‑Designing a Pre‑Radiotherapy Exercise Programme With People Living With Lung Cancer

**DOI:** 10.1111/hex.70837

**Published:** 2026-08-26

**Authors:** Matthew Beggs, Joanne Reid, Gerard G Hanna, Abbie Edgar, Seamus Cotter, David Burke, Gillian Prue

**Affiliations:** ^1^ School of Nursing and Midwifery Queens University Belfast Belfast Northern Ireland; ^2^ Discipline of Radiation Therapy Trinity College Dublin Ireland; ^3^ Department of Radiation Oncology St. Luke's Radiation Oncology Network Dublin Ireland; ^4^ The Thomas J Moran Graduate School Queen's University Belfast Belfast Northern Ireland; ^5^ Irish Lung Cancer Community (ILCC) Dublin Ireland; ^6^ Johnston Cancer Research Centre Queen's University Belfast Northern Ireland

**Keywords:** co‐design, complex intervention, exercise, intervention development, lung cancer, oncology, patient and public involvement, patient participation, physical activity

## Abstract

**Background:**

Emerging evidence suggests exercise may improve radiotherapy efficacy by reducing treatment resistance caused by tumour hypoxia. However, recruitment to exercise oncology studies is low (median 38%), particularly for people with lung cancer. One strategy to address such engagement barriers is by collaborating through Patient and Public Involvement and Engagement (PPIE).

**Objective:**

To co‐design an acceptable pre‑radiotherapy exercise programme for people living with lung cancer, incorporating the perspectives and experiences of those affected by the disease.

**Methods:**

A PhD researcher collaborated with people impacted by lung cancer and an expert advisory group in a series of meetings and workshops underpinned by the principles of experience‐based co‐design and Boyd's co‐design approach. Workshops were recorded and transcribed verbatim and analysed using reflexive thematic analysis. Findings relating to programme design were deducted into Frequency, Intensity, Time and Type principles, whereas barriers and facilitators were deducted into the five domains of the Practical planning for Implementation and Scale‐up guidelines.

**Results:**

Seven attendees accepted workshop invitation, of which three had received a lung cancer diagnosis, and four were family members. Considering design, daily exercise was regarded acceptable for short duration treatment plans, with an optimal duration of 20 min. Exercise intensity must also be personalised and autoregulated. Furthermore, 15 barriers and 40 facilitators were identified, the most pertinent relating to physical activity coach conduct and research design. For example, supervised exercise and developing professional working relationships were considered essential.

**Conclusions:**

This project shows how PPIE can effectively inform early research to improve acceptability and presents the first co‑designed pre‑radiotherapy exercise programme for lung cancer.

**Patient or Public Contribution:**

PPIE was integral for this project. People diagnosed with lung cancer, along with family members, participated in two co‑design workshops, contributing their perspectives and experiences to shape the development of a pre‑radiotherapy exercise programme. Patient advocates within the advisory group reviewed and refined workshop materials to ensure they were accessible, meaningful, and patient‑friendly. Finally, one co‑design attendee and a patient advocate from the advisory group assisted drafting this manuscript to ensure published findings authentically represent the workshop discussions and the lived experiences shared throughout the project.

**Abbreviations**
AG: advisory group
DHSC: Department of Health and Social Care
EBCD: experienced based co‐design
FITT: frequency intensity time type
GRIPP2: Guidance for Reporting Involvement of Patients and the Public
HCP: healthcare professional
HIIT: high‐intensity interval training
ILCC: Irish Lung Cancer Community
LC: lung cancer
NICRF: Northern Ireland Cancer Research Consumer Forum
NSCLC: non‐small cell lung cancer
PPIE: Patient and Public Involvement and Engagement
PRACTIS: PRACTical planning for Implementation and Scale‐up
RT: radiotherapy

## 1 Background

Within the past 15 years, an accumulating body of evidence highlights the value of introducing exercise across the cancer continuum to improve patient outcomes [1, 2, 3, 4]. In lung cancer (LC), emerging preclinical evidence suggests exercise may enhance treatment efficacy through mechanisms such as improved tumour oxygenation and increased immune cell infiltration [5, 6, 7]. This is particularly relevant to radiotherapy (RT), as tumour hypoxia can occur in 50%–80% of non‐small cell lung cancers (NSCLC), making tumours two to three times more resistant to RT [6]. Given LC's status as the leading cause of cancer‐related mortality with poor survival outcomes [8, 9, 10, 11], investigating exercise as a strategy to improve outcomes remains a research priority [12, 13]. However, recruitment to exercise oncology studies is challenging. A recent systematic review found a median recruitment rate of 38% across 86 exercise oncology trials not limited by date, citing ‘*patient‐centric’* barriers as the most significant limitation [14]. This is particularly relevant for people living with LC who experience significant challenges with exercise adherence [13, 14, 15], resulting in limited recruitment to exercise trials [10, 16, 17] with high attrition rates [12, 18, 19, 20]. These barriers result in low‐quality evidence and significantly hinder progress towards high‐quality efficacy trials and optimal exercise prescriptions [13, 21].

One strategy to address these barriers is by collaborating with stakeholders through Patient and Public Involvement and Engagement (PPIE) [14]. PPIE has expanded significantly in healthcare research over the past decade, reflecting the growing emphasis on person‐centred care [19, 20, 22]. Similarly, co‐design, an approach involving equal partnership between stakeholders and researchers, has become an internationally recognised method in healthcare research [23, 24, 25, 26]. While related, PPIE and co‐design differ: PPIE shapes research design and conduct to improve acceptability, whereas co‐design generates data to address research questions [27, 28, 29, 30]. However, underpinning PPIE with co‐design methods has been successfully utilised to improve the acceptability of LC services and research initiatives [25, 28, 31, 32], including increasing screening uptake [33] and developing multi‐modal rehabilitation programmes [34]. Therefore, this project aims to engage stakeholders using PPIE underpinned by co‐design methodology to co‐create a pre‐radiotherapy exercise programme for people living with LC, optimising recruitment and adherence for future feasibility testing. Within the context of this paper, pre‐radiotherapy exercise refers to the performance of exercise throughout treatment, performed within the RT department, immediately before each RT administration.

## 2 Methods

### 2.1 Introduction

This project was conducted in accordance with principles of Boyd's co‐design approach [25, 32], which was developed upon six key elements to better understand the patient experience: (1) Engage, (2) Plan, (3) Explore, (4) Develop, (5) Decide, and (6) Change. The project also adhered to the structures of accelerated experience‐based co‐design (AEBCD), with modification. Experienced‐based co‐design (EBCD) is a narrative‐based approach to co‐design which collaborates lived experience with the experience of healthcare professionals (HCP) through the hosting of individual and collaborative workshops. Before workshop hosting, EBCD recommends the conduct of video interviews of people with lived experience to inform workshop discussions. AEBCD replaces interviews with readily available, pre‐recorded videos when resources are limited. However, considering the novelty of a pre‐radiotherapy exercise intervention, publicly available videos discussing this subject were limited. As an alternative approach, a scoping review [35] was conducted to understand the evidence and inform workshop discussions. Similarly, evidence‐based approaches have been exemplified within the literature [36, 37]. Finally, reporting of this project aligns with the Guidance for Reporting Involvement of Patients and the Public (GRIPP2) Short Form Checklist [27] (see Supplementary File S1).

### 2.2 Advisory Group

An Advisory Group (AG) was established to oversee the PPIE project. Dependent on AG member availability, AG meetings could be conducted as either group or individual sessions before and after each workshop to plan and discuss workshop outcomes. This process facilitated bidirectional communication between HCPs and workshop attendees as collaborative partners. All AG meetings were facilitated by M.B. AG membership was guided by predefined criteria (Table 1). In total, 12 members joined the AG, and their characteristics and relevant experience is summarised in Table 2.

**Table 1:** Advisory group desired criteria.

*Inclusion criteria*
Aged 18 years or over
Willing and able to participate and provide input into pre‐ and post‐workshop meetings
Has experience in the management/facilitation of radiotherapy treatment for people with LC OR
Has experience in the facilitation or prescription of exercise and physical activity for people living with cancer OR
Is actively involved in cancer or exercise research
*Exclusion criteria*
Is not involved in cancer care (either as an academic/research role or working directly with patients)
Has little or no experience with co‐design and/or patient and public involvement.

**Table 2:** Advisory group members.

AG member	Relevant experience
Two academics lecturers	An Associate Professor and a Lecturer, both experienced in cancer exercise research
Two oncologists	A Consultant Clinical Oncologist specialising in the treatment of lung, head and neck, and skin cancers, alongside an Academic Clinical Lecturer and Specialist Registrar experienced in oncological care and leading cancer exercise research care and leading cancer exercise research
Chairperson of an LC support charity (Patient Advocate)	Personal experience with LC and patient advocate among PPI groups and international conferences
A LC nurse consultant	40 + years’ experience in nursing with 30 + years specialising in palliative care
Two LC nurse specialists	Years experience working as clinical nurse specialists, managing care for people living with LC
A LC specialist radiographer	A band 8a therapeutic radiographer with years’ experience specialising in LC care
Two senior radiographers	Research and development radiographer with years’ experience supporting cancer trials and a senior therapeutic radiographer
A senior physiotherapist	Senior physiotherapist and Assistant professor specialising in cancer prehabilitation, rehabilitation and the role of physical activity in cancer survivorship.

### 2.3 Stakeholder Criteria

The following key stakeholder criteria was established to support the aims of this PPIE project.

#### 2.3.1 People Living With Lung Cancer

People living with LC were invited to attend the co‐design workshops based on their experience living with the disease and receiving treatment. The following desirable criteria (Table 3) were established to ensure diversity among invited attendees, whilst limiting ineligible involvement.

**Table 3:** Desired criteria.

**Desired criteria: People living with lung cancer**	**Desired criteria: Family and informal caregivers**
Aged 18 years or over	Aged 18 years or over
Willing to participate in online workshops	Willing and able to participate in online workshops
A LC diagnosis and either completed or receiving treatment	Family member or an informal caregiver to a person who has received a LC diagnosis
Able to understand and communicate in English	Able to understand and communicate in English.
**Exclusion criteria**	**Exclusion criteria**
Is a resident outside of the UK or Ireland	Lived experience, knowledge or skills was not applicable to LC

#### 2.3.2 Family or Informal Caregivers

As the impact of LC and its treatment extends beyond the individual [38, 39, 40], invitations were extended to informal caregivers or family members (Table 3). Within this project, informal caregivers were defined by the Department of Health and Social Care (DHSC) as ‘Someone who provides unpaid help to a friend or family member needing support’ [40]. Although a UK definition of carer was adopted, it remained consistent the definition used in Ireland [41].

### 2.4 Invitation Strategy

M.B initially consulted a regional cancer PPI group, the Northern Ireland Cancer Research Consumer Forum (NICRCF), to obtain feedback and identify strategies for workshop promotion. As the project was led in Northern Ireland, extending workshop invitation across the island of Ireland was recommended. This approach aligns with the all‐island commitment of the Patient and Public Involvement (PPI) Ignite Network and Queen's University Belfast (QUB) to improve PPI accessibility [42, 43].

Attendees were subsequently invited through multiple channels, including online dissemination via six cancer charities and six PPI groups; poster distribution across six local healthcare settings; public poster dissemination in seven local charity shops; engagement with two local cancer exercise groups; university communication networks; and social media platforms. In addition, a public information event was hosted online. Individuals who expressed interest were invited to attend a follow‐up discussion via telephone or Microsoft Teams to discuss detailed information on workshop involvement.

### 2.5 Setting

As recommended by the NICRCF, co‐design workshops were conducted online via password‐protected Microsoft Teams meetings to minimise travel requirements and facilitate participation across the UK and Ireland. All attendees were offered an individual online session before each workshop to address technical issues.

### 2.6 Methods of Engagement

Two linked co‑design workshops were prepared by M.B (Table 4). As recommended by Boyd [32], a workshop summary sheet was completed and agenda emailed to attendees before each workshop (Online Supplementary Material S2 and S3). Workshop were video and audio‑recorded by the facilitator with verbal consent from all attendees. All recordings were deleted following transcription. AG meetings were not audio‐recorded or transcribed. Instead, M.B completed meeting summary sheets to capture key recommendations. As the AG functioned in a steering capacity only to ensure that clinical recommendations and safety considerations were reflected in co‐design discussions, AG summary sheets were not included in deductive analysis.

**Table 4:** Co‐design workshop details.

	Workshop 1	Workshop 2
Duration and setting	2 h (online)	2 h (online)
Focus	1. To co‐design an initial draft of the pre‐radiotherapy exercise programme	1. To confirm and make final amendments to the pre‐radiotherapy exercise programme 2. To explore barriers and discuss solutions to potential problems that may be encountered throughout the conduct of a future study
Aim	− To explore the experience of people with lung cancer engaging in exercise/physical activity. − To discuss components of the pre‐radiotherapy exercise design, including frequency, intensity, time, and type. − To discuss potential areas of support which could be implemented to improve the participant's experience	− To discuss, and finalise an agreement on the design of the pre‐radiotherapy exercise programme − To complete a Participant Journey Mapping workshop activity [32] to discuss the potential barriers that may be encountered throughout the study at the following stages: Diagnosis, treatment planning, first day of treatment, during treatment, and treatment completion. − To discuss any ideas or concerns raised by attendees which has not been previously covered throughout the workshops.
Techniques	− Icebreakers − Presentation − Roundtable discussion	− Icebreakers − Presentation − Roundtable discussion − Patient journey mapping
Main activity	1. Introductions and icebreaker 2. Initial presentation 3. RT discussion 1: Exercise history and experience 4. RT discussion 2: Elements of a pre‐radiotherapy exercise programme 5. RT discussion 3: Methods of providing support to improve people with LC experience with exercise	1. Introductions and Icebreaker 2. Initial presentation (summarising WK 1 findings) 3. RT discussion 1: draft exercise programmes 4. Patient journey mapping exercise
Post‐workshop activities, including data analysis and interpretation	− Audio transcription − Reflexive thematic analysis − Creation of draft pre‐radiotherapy exercise programmes − Discussions with advisory group − Presentation preparation for workshop two (providing summary of key points)	− Audio transcription − Reflexive thematic analysis (Deductive approach into FITT principles and PRACTIS Domains − Final draft of pre‐radiotherapy exercise programme created − Presentation of findings to AG for final discussion
Expected outputs	− Insights into people with LC's thoughts and experiences regarding exercising in general and throughout treatment − Initial ideas for a pre‐radiotherapy exercise programme design − Initial discussions on potential strategies to improve recruitment and adherence of people with LC to exercise programmes	− Final draft of pre‐radiotherapy exercise programme − Identified key strategies to facilitate a patient‐centred exercise programme, which can improve recruitment and adherence − Identified key barriers and facilitators

#### 2.6.1 AG Meeting 1

AG meeting 1 was hosted to understand initial thoughts from HCPs, focusing specifically on treatment pathways and patient care needs, which must be considered within the exercise programme design. The latest evidence was discussed, and session plan drafted to inform Workshop 1 discussions.

#### 2.6.2 Workshop 1

Workshop 1 aimed to create an initial draft of the exercise programme. The session began with an introductory activity to foster engagement, followed by a brief presentation outlining the agenda and topics. Attendees then engaged roundtable discussion one, exploring the perspectives of people with LC engaging on exercise. The second discussion focused on programme design structured according to the FITT principles—frequency, intensity, time, and type [44]. To guide discussions, attendees considered the pre‐radiotherapy exercise programme from Egegaards’ study [45] as an example. Once consensus was reached, attendees discussed strategies to improve the exercise experience of people with LC during cancer treatment. The workshop concluded with a summary of key points and verification from attendees to ensure accuracy.

#### 2.6.3 AG Meeting 2

AG meeting 2 was hosted to discuss Workshop 1 outcomes. In preparation, each AG was e‐mailed a summary sheet for review. During AG2 meetings, a session plan for Workshop 2 was drafted, including four potential exercise programmes to be presented.

#### 2.6.4 Workshop 2

Workshop 2 aimed to agree the final draft of the exercise programme and identify potential barriers and facilitators. In preparation, attendees were sent the drafted exercise programmes for review.

The Workshop began with an introductory activity, a brief presentation revisiting key points from Workshop 1, and a summary of AG 2 discussions. Attendees then engaged in the first roundtable discussion to critically discuss the pre‐prepared exercise programmes. This was followed by a participant journey mapping exercise [32, 46], where attendees considered the hypothetical patient journey across five phases: diagnosis and recruitment, treatment planning, initiation of treatment and exercise, maintenance during treatment, and post‐treatment follow‐up. For each phase, participants identified potential emotional responses and barriers to exercise engagement, then proposed and prioritised solutions. The workshop concluded with a summary of key points and confirmation from attendees to ensure accuracy.

#### 2.6.5 AG Meeting 3

AG meeting 3 was hosted to discuss workshop 2 outcomes. This included confirmation of the final exercise programme, making any adjustments to assure patient safety if necessary. Logistics were also considered to ensure the programme could be practically implemented within a local radiotherapy department and was congruent with standard treatment pathways.

### 2.7 Information Interpretation

Workshop recordings were transcribed verbatim by the facilitator and managed using Microsoft Word and NVivo (version 12). Transcripts were then reviewed following the principles of Braun and Clarke's six‐step approach to reflexive thematic analysis [47, 48]. As all information from workshop activity notes was captured in the transcripts, these were excluded from the review. A deductive approach was then applied to categorise all identified information into two predetermined frameworks selected for their international recognition and application to exercise oncology:

- The FITT principle—an internationally recognised and validated method of exercise prescription [4, 44, 49].
- The PRACTIS Guide—a framework for implementing physical activity research into clinical practice [50].

M.B. undertook initial line‐by‐line screening of transcripts to identify outcomes relevant to each framework. Following identification, outcomes were subsequently coded, grouped into themes, and deductively categorised within the relevant framework. Identified codes, themes and categorisations were independently reviewed by two members of the research team (GP and JR) to refine coding and verify categorisations. In the event of a disagreement, three members of the research team (MB, GP, and JR) discussed the issue until consensus was reached.

#### 2.7.1 FITT Principles

The FITT principles provide a standardised format of ‘planned exercise prescription’ [49], enabling systematic monitoring of adherence to key components. Accordingly, outcomes on programme design were deducted within the following categories:

− Frequency: How often to run exercise sessions
− Intensity: Difficulty of the exercise session
− Time: Each session and entire programme duration
− Type: The types of exercise involved

#### 2.7.2 Barriers and Facilitators: PRACTIS Guidelines

Barriers and facilitators identified during workshop discussions were categorised using the five domains of the PRACTIS guide [50]. These include:

1. **Implementer Characteristics**—Attributes of those delivering the intervention, including skills, experience, attitudes, and motivations.
2. **Delivery Setting/Organisational Characteristics**—Contextual factors such as leadership, capacity, structural features, and workplace culture.
3. **Community Characteristics**—Broader influences like funding, policy, political climate, and competing priorities.
4. **Intervention Characteristics**—Core design elements, including complexity, evidence quality, cost, adaptability, and alignment with organisational values.
5. **Process of Implementation**—Factors affecting delivery, including target population characteristics, recruitment accessibility, and engagement from providers and participants.

#### 2.7.3 Reflexive Statement

I (M.B.) am a white male living in Northern Ireland, with a professional background as an NMC registered nurse with 6 years’ experience working in acute cancer services. Additionally, I have maintained a strong personal and professional interest in exercise. This interest sparked a critical question: Could exercise benefit the people living with cancer in my care? This curiosity led me to pursue an MSc exploring the potential benefits of exercising throughout chemotherapy. Concurrently, I pursed professional exercise qualifications, including level 3 personal training and Level 4 Cancer Exercise Specialist.

The combined experience of cancer nursing, academic inquiry, and specialist exercise training has shaped my belief that exercise can play a meaningful role in cancer care, While I acknowledge that this belief drives my professional motivation, I recognise its potential for incurring bias when facilitating AG meetings and co‐design workshops.

This risk was reduced through regular consultation with AG, who contributed to the design of workshop activities and reflected upon workshop discussions. Furthermore, outcomes were shaped by the expert insights of co‐design attendees, who acted as collaborative partners. In addition, the remaining authors provided oversight during manuscript preparation, ensuring project outcomes remained central to the attendee's perspectives.

### 2.8 Ethics Statement

According to the PPI Ignite Network, ethical approval is not required for involvement in research planning, design, interpretation, or dissemination [51]. Therefore, ethical notification was not required for this project as confirmed by QUB Research Governance Department. All contributors were fully informed through written communication and a pre‐workshop meeting, where questions could be addressed. Attendance was voluntary, with the option to withdraw at any time. All contributors provided verbal consent and confirmed via email that they were willing to engage in the co‐design workshops. To acknowledge contributions, attendees received an online gift voucher for each workshop attended.

### 2.9 Information Management

As this project was conducted as PPIE, discussions were processed as collective outcomes rather than research data. Consequently, a formal Data Protection Impact Assessment (DPIA) was not required. However, the documentation and storage of outcomes strictly adhered to QUB's information management policy. Accordingly, all workshop materials, attendee information, and transcripts were stored securely within a QUB One Drive account. Access to this information was restricted to key team members, and all shared outputs were de‐identified. This ensured the collaborative outputs were recorded accurately and stored securely in compliance with institutional standards.

## 3 Outcomes

The outcomes sections have a two‐part structure: First, a summary of attendee characteristics is provided, followed by a brief description of the workshop's arrangements. Next, the co‐designed exercise programme is presented, followed by a discussion of the identified barriers and facilitators.

### 3.1 Characteristics

As formal ethical approval was not required, attendees engaged as collaborative partners rather than research subjects. Consequently, Socioeconomic Group (SEG) and Social Determinants of Health (SDOH) data were not collected. Only non‐identifiable characteristics were retained and presented in Table 5 for context.

**Table 5:** Attendee characteristics and workshop attendance.

Currently receiving LC treatment	Completed LC treatment	Friend or family member	Gender	Age		Workshop attendance	
				18–49	50+	Wk1	Wk2
Attendee 1	**—**	**—**	Female	**—**	•	•	**—**
**—**	Attendee 2	**—**	Male	**—**	•	•	•
**—**	**—**	Attendee 3	Female	**—**	•	•	•
**—**	**—**	Attendee 4	Female	•	—	•	**—**
Attendee 5	**—**	—	Female	**—**	•	•	•
**—**	**—**	Attendee 6	Female	•	**—**	•	•
**—**	**—**	Attendee 7	Female	•	**—**	**—**	•

In total, 7 attendees accepted the invitation. Most attendees were female (6 of 7), aged 50 years or over (4 of 7), and were a family member of a person who had been affected by LC (4 of 7). No attendees contributing to workshops were related. Two attendees were currently receiving LC treatment, and one attendee had completed LC treatment but had previously received RT, all who reported attending a local exercise group at the time of discussion. Attendee characteristics and workshop attendance is provided in Table 5.

### 3.2 Workshop Arrangement

Due to logistical constraints, scheduling a single date that accommodated all attendees was not feasible. Consequently, each workshop was repeated on separate dates to maximise participation. When only one attendee was available, a condensed 1‑h session was delivered.

Although workshops scheduling was flexible, one attendee declined attending workshop 1, and two attendees declined attending Workshop 2. As workshop participation was voluntary, reasons for non‐attendance were not recorded. Furthermore, individuals who declined workshop attendance were not contacted to provide input through alternative methods, such as telephone interviews or email correspondence.

**Figure 1:**
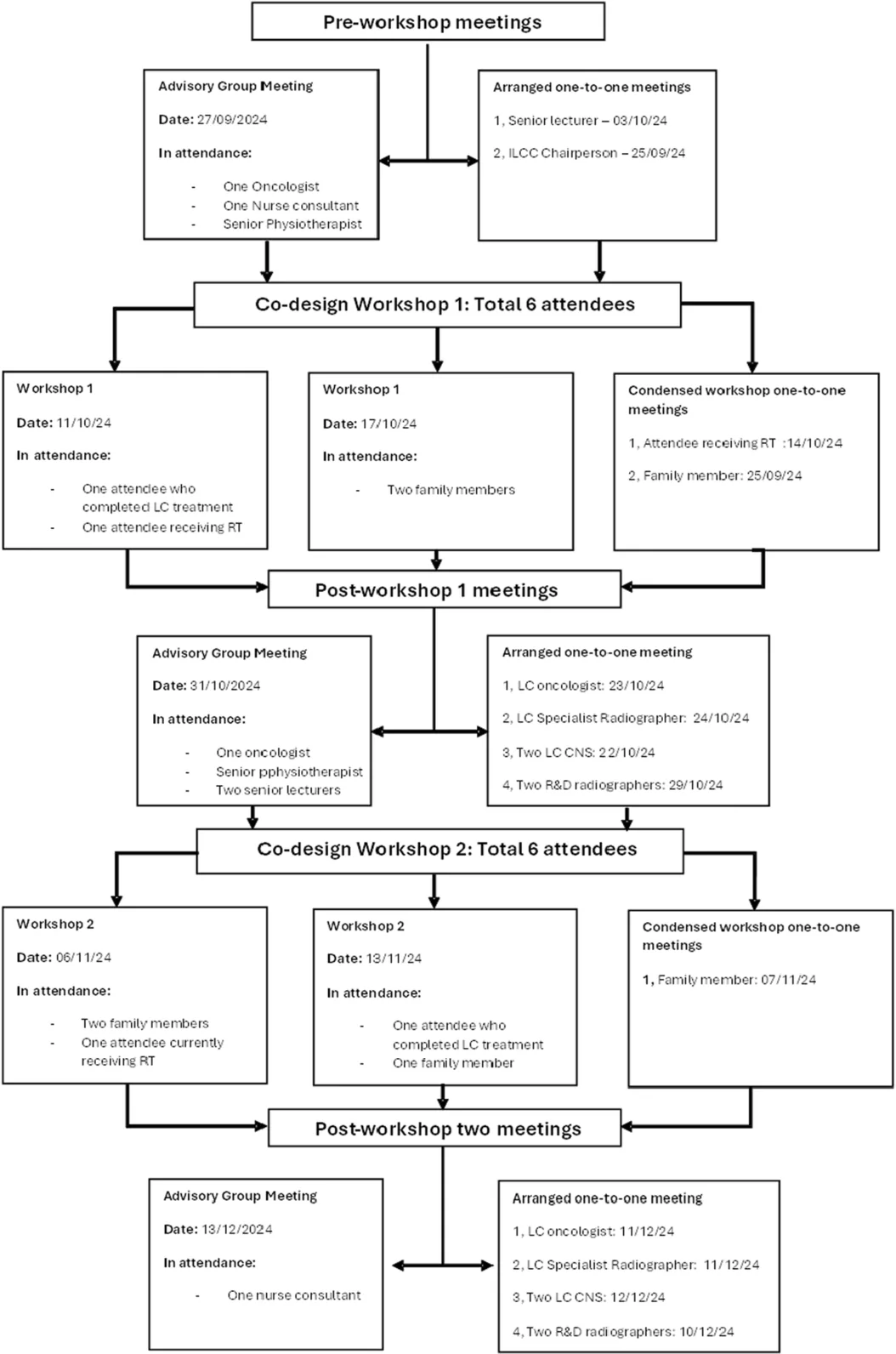
AG and Workshop dates and attendance flow chart.

The dates and structure of all AG and co‑design workshops are presented in Figure 1.

### 3.3 The Co‐Design Exercise Programme

#### 3.3.1 Frequency

Attendees emphasised the importance of a clearly defined exercise programme with a definite start and end, recommending a short duration of 2–5 weeks. They noted this limited timeframe reassures participants that the increased physical demands are temporary. Consequently, there was unanimous agreement that exercising 5 days per week, over a 4‐week period, is practical and feasible, whereas daily commitments over longer periods were considered discouraging.

#### 3.3.2 Intensity

Attendees concluded that all exercise intensities should be personalised based on baseline fitness and characteristics such as age. To achieve this, attendees recommended incorporating multiple intensity levels, progressing in difficulty, to enable adjustment based throughout the programme. This flexible approach may make the study more appealing to participants. However, coaches should frame progression as ‘aims’ rather than ‘targets’ to avoid creating a sense of failure.

Attendees also advocated for high‐intensity exercise, which was reported as ‘enjoyable’, associated with a ‘sense of accomplishment’. However, excessive high‐intensity may discourage participation, and should only be encouraged when participants feel comfortable engaging in it.

#### 3.3.3 Time (Duration)

Attendees agreed that exercise duration should long enough to provide benefits and a sense of accomplishment, yet short enough to avoid overwhelming participants. Attendees agreed that a 20‐min duration (including warm‐up) was ideal, particularly if performing five sessions per week. However, duration may be adjusted based on individual fitness and preferences.

#### 3.3.4 Type

Attendees discussed a range of exercise options, from treadmill walking to Tai Chi. Five of seven attendees favoured stationary rowing for its ability to engage both upper and lower body muscle groups and its perceived enjoyment. In contrast, cycling on a stationary bike elicited mixed responses, although acknowledged as a simple option with easily adjustable intensity. Offering a variety of exercise types to accommodate participants’ preferences was suggested, although subject to resource availability. However, when selecting exercises, personal safety was emphasised, particularly balance, as people living with LC often experience unstable mobility. Therefore, seated or low‐to‐the‐ground exercises should be prioritised.

**Figure 2:**
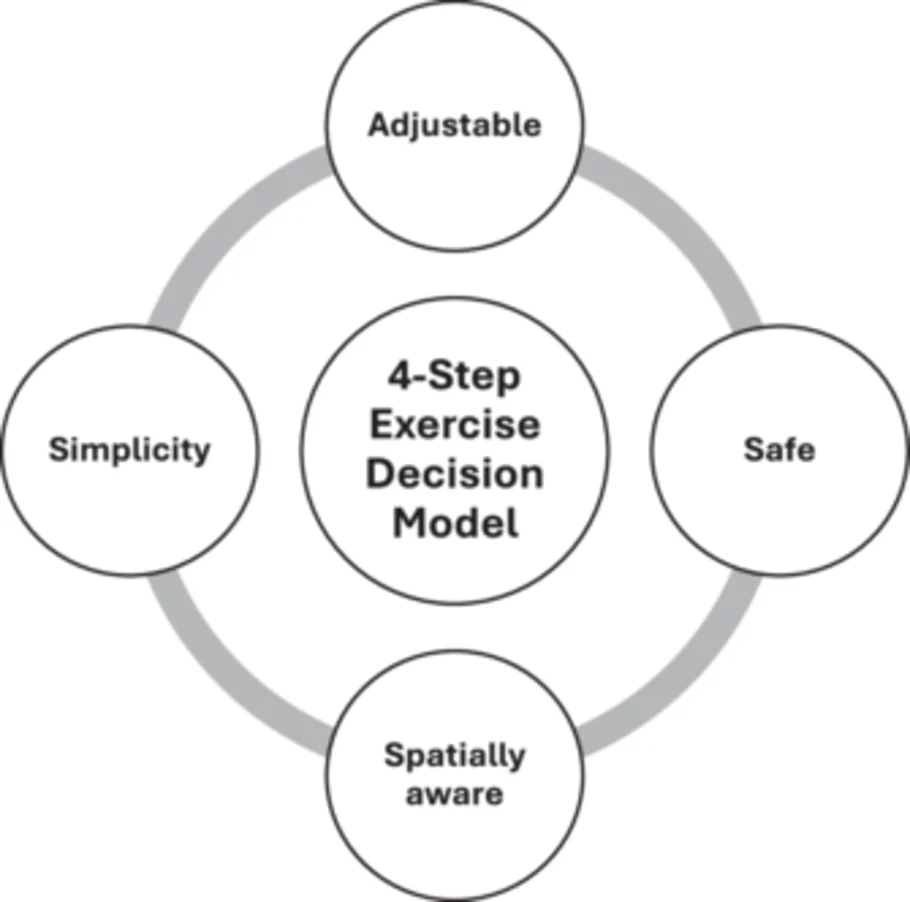
Four‐step exercise decision model.

In conclusion, a 4‐step model (Figure 2) was created, consisting of four principles which should be considered when choosing an exercise:

### 3.4 Deductive Analysis Framework 1: PRACTIS Guidelines: Barriers and Facilitators

Attendees identified 15 barriers and 40 facilitators (Table 6). In some instances, barriers and facilitators were sub‐categorised within their respective domains when multiple factors related to a similar concept. Although discussions focused on a pre‐RT exercise programme, the following barriers and facilitators are likely applicable to exercise oncology research more broadly.

**Table 6:** PRACTIS domains: Deducted barriers and facilitators identified during co‐design.

PRACTIS Domains									
Implementer characteristics		Delivery setting/organisation characteristics		Community characteristics		Intervention characteristics		Process of implementation	
Barriers (*n* = 3)	Facilitators (*n* = 10)	Barriers (*n* = 1)	Facilitators (*n* = 3)	Barriers (*n* = 2)	Facilitators (*n* = 1)	Barriers (*n* = 5)	Facilitators (*n* = 13)	Barriers (*n* = 4)	Facilitators (*n* = 13)
Coach motivational incompetencies (**2**)	*PAC conduct*: **‐** Developing a professional, working relationship (10) **‐** Personalised coaching style (2) **‐** Understanding individual capabilities (**1**) **‐** Belief in participants capabilities (1) **‐** Foster a positive and welcoming attitude (3)	Limited Resources (**5**)	Integration with radiotherapy treatment plan (**7**)	Geographical location (**2**)	Considering the Socio‐cultural context (**2**)	Insufficient programme design (**2**)	*Personalisation*: ‐ Adaptable exercise programme (**9**) ‐ Collaborative goal setting (**3**) ‐ Incorporate personalised elements (**20**)	*Symptoms and side effects:* ‐ Radiotherapy side‐effects (**6**) ‐ Falls risk (**2**)	*History, experience, and perception of exercise and research:* ‐ Previous experience of physical activity (**3**) ‐ Beneficial perception (**2**) ‐ Positive perception of research (**1**)
A coach lacking self‐awareness (**2**)	*Acknowledgement and gratitude*: ‐ Tracking and Celebrating Achievements (**19**) ‐ Express gratitude (**2**)		Creating a supportive environment (**6**)	Traffic (**1**)		Lack of purpose and benefits (**2**)	*Design specific:* ‐ Delaying study presentation (**3**) ‐ Appropriately timing follow‐up interviews (**14**) ‐ Purpose‐driven program (**22**) ‐ Use of inclusive terminology (**8**) ‐ Flexible exercise start time (**2**)	*Mental health:* ‐ Fear, stress and anxiety (**12**)	***2.** Research Team Support:* ‐ Having a nominated contact (**3**) ‐ Design and provision of written study documents (7) ‐ Provision of written educational material (**6**) ‐ Provide psychological support (**7**) ‐ Autoregulation (**1**) ‐ Provide additional time for emotional support during key treatment dates (2) ‐ Introduce a preparation session (23)
Authoritative coaching style (**3**)	*Incorporate teaching and education throughout:* ‐ Facilitating physical activity education (**13**) ‐ Explanation of assessments and study material (**4**) ‐ Raise awareness of treatment side effects (**1**)		In‐hospital exercise sessions (**1**)			Presentation at diagnosis (**9**)	*Exercise supervision* (**4**)	History, experience, and perception of exercise (**13**)	Supportive relationships (**23**)
						Group exercise (**3**)	*Enjoyment and Socialisation:* ‐ Incorporate elements of competition (5) ‐ Integrate music into exercise sessions (**4**) ‐ Incorporate variable intensities (**1**) ‐ Incorporate socialisation (**6**)		*Study Presentation:* ‐ Oncologist conducts initial study presentation (**2**) ‐ Alternative Healthcare Professionals (**2**)
						Purchase of additional exercise equipment (**1**)			

Abbreviations: *n*, total number of barriers/facilitators with the domain; (1), number of references in transcripts.

#### 3.4.1 Domain 1: Implementer Characteristics

##### 3.4.1.1 Barriers

Attendees acknowledged that inadequate encouragement may undermine adherence, particularly among individuals requiring external support to overcome physical and psychological barriers. However, they stressed that encouragement should never become coercion, and forceful language (e.g., ‘You must’) risks appearing authoritative and discouraging. Instead, a collaborative approach fosters engagement and avoids perceptions of feeling ‘bullied.’

##### 3.4.1.2 Facilitators

Facilitators were sub‐categorised into three themes:

1. PAC conduct
2. Acknowledgement and gratitude of participants involvement
3. Incorporate teaching and education into the programme design

1. Physical activity coach conduct
  PAC conduct was recognised as a key facilitator, as attendees perceived coaches as leaders who can influence feasibility outcomes by fostering positive yet professional relationships. Furthermore, a PAC should understand and demonstrate belief in an individual's capabilities and maintain an open, welcoming attitude. One attendee highlighted the value of humour to create a positive environment. Through this approach a PAC can identify when participants are exercising outside their capacity to enable appropriate adjustments to preserve safety and prevent feelings of failure.
  Attendees also noted that preferences for encouragement vary. some participants may appreciate frequent verbal support, while others may prefer minimal input. Consequently, developing professional relationships and understanding individual differences is essential for tailoring motivational strategies to personal preferences.
2. Acknowledgement and gratitude
  Attendees emphasised the importance of recognising individual achievements to sustain motivation. Suggested strategies included tracking progress (e.g., weekly cycling distance), documenting pre‐ and post‐exercise reflections to support feedback and self‐reflection, and creating a communal ‘quote board’ to anonymously foster encouragement. Acknowledging participants’ physical effort as an expression of gratitude was also emphasised by providing personalised thank‐you cards or certificates of achievement upon completion.
3. Incorporate teaching and education throughout
  Several attendees (2,3,5) highlighted the value of leveraging the study as an educational platform to promote the benefits of exercise. Furthermore, a PAC should clearly explain any assessments or study materials before use to minimise confusion or anxiety. Finally, coaches should have a basic understanding of RT side effects and their implications for exercise participation as the increased contact may facilitate the early identification of side effects requiring medical intervention.

#### 3.4.2 Domain 2: Delivery Setting or Organisation Characteristics

##### 3.4.2.1 Barriers

Potential barriers related to the exercise setting included restricted space within the RT department, limited healthcare staff to support the study, and limited availability of exercise equipment to accommodate participant preferences.

##### 3.4.2.2 Facilitators

Collaboration with the lung multidisciplinary team to align exercise with RT schedules was a recognised facilitator to minimise travel‐related barriers and leverage hospital waiting periods. Furthermore, integrating exercise into treatment plans may normalises exercise as part of routine care. Additionally, hospital‐based sessions were perceived as safe and reassuring due to the presence of HCPs and hospital facilities.

#### 3.4.3 Domain 3: Community Characteristics

##### 3.4.3.1 Barriers

Geographical location was identified as a barrier to exercise participation, particularly people living in rural areas. Furthermore, reliance on self‐transport was suggested to become increasingly difficult as treatment‐related fatigue increased. Therefore, scheduling additional hospital visits for exercise can significantly increase burden.

##### 3.4.3.2 Facilitators

Attendees noted that incorporating culturally appropriate exercise modalities could enhance participation. As noted by Attendee 1, tailoring programmes to culturally relevant activities may improve engagement.

#### 3.4.4 Domain 4: Intervention Characteristics

##### 3.4.4.1 Barriers

Attendees overwhelmingly agreed that participants should not be approached at the point of diagnosis, as this is a highly stressful period. Attendees also noted that exercise programmes lacking a clear purpose or those that were insufficiently stimulating to yield meaningful benefits (e.g. improving mental health and supporting activities of daily living), may discourage engagement.

Additionally, exercise programmes not perceived as enjoyable, or require the personal purchase of equipment, may discourage involvement. Finally, attendees expressed concerns that group‐based exercise may deter individuals with low baseline fitness due to feelings of embarrassment.

##### 3.4.4.2 Facilitators

Facilitators were sub‐categorised into four themes:

1. Personalisation
2. Exercise programme design
3. Exercise supervision
4. Enjoyment and socialisation

1. Personalisation
  Attendees emphasised the importance of incorporating personalisation through collaborative goal setting based on individual capabilities and preferences, rather than a ‘one‑size‑fits‑all’ approach. Personalisation may also mitigate feelings of insignificance as some individuals experience in healthcare, such as ‘feeling just like a number’. However, attendees acknowledged that resource limitations could constrain the extent of personalisation.
2. Exercise programme design
  Attendees recommended presenting the exercise programme at least 1 week after diagnosis to allow sufficient time for individuals to process their diagnosis. However, one attendee suggested that individuals may be most receptive at diagnosis, as people often conduct their own research immediately following diagnosis. Regardless, when presenting the study, attendees advised avoiding terminology such as ‘intervention’ and ‘intensity’, as these terms can appear clinical and intimidating. Instead, more inclusive language, such as ‘physical activity programme’ and ‘effort’, was recommended. Attendees also proposed coordinating with RT staff to allow a flexible 5–10‐min start window for all exercise sessions.
3. Exercise Supervision
  Attendees discussed the potential impact of supervised and unsupervised sessions. While some suggested unsupervised sessions could foster confidence, others raised safety concerns and advocated for fully supervised sessions only. Attendees also noted that low confidence could deter participation if unsupervised. Ultimately, attendees agreed that the PAC plays a critical role in ensuring exercise safety. As a result, participants may feel more confident exercising under supervision and was recommended to maximise engagement.
4. Enjoyment and socialisation
  Attendees discussed strategies to make exercise programmes more enjoyable. These included incorporating variable exercise intensities, and introducing friendly competition between participants (e.g., displaying a ‘highest score’). They also suggested integrating music into sessions by encouraging participants to bring headphones or playing their preferred music. Furthermore, attendees recommended adding social elements, such as group exercise or regular social events (e.g. coffee mornings) to enhance engagement.

#### 3.4.5 Domain 5: Process of Implementation

##### 3.4.5.1 Barriers

Barriers were sub‐categorised into three themes:

1. Symptoms and side effects
2. Mental health
3. History, experience, and perception of exercise

1. Symptoms and side‐effects
  As RT side effects intensify, such as fatigue, exercising may become increasingly challenging as treatment progresses, particularly among older participants.
2. Mental Health
  Attendees noted the stress of a cancer diagnosis, combined with starting treatment may reduce willingness to participate. For some individuals, introducing exercise during treatment may exacerbate anxiety and reduce their capacity to self‐motivate and engage with exercise, especially if experiencing side‐effects.
3. History, experience, and perception of exercise
  Attendees recognised that participants who have had a negative experience with exercise or are unaware of its benefits may not prioritise it and focus on treatment. Conversely, some may associate exercise with high intensity only, discouraging involvement. Moreover, people with a recent history of sedentary behaviour may perceive themselves as too unfit for exercise.

##### 3.4.5.2 Facilitators

Facilitators were sub‐categorised into four themes:

1. History, experience, and perception of exercise and research
2. Research team support
3. Peer support
4. How the study is initially presented

1. History, experience, and perception of exercise and research
*Previous experience of physical activity*
  Attendees noted that those with positive exercise experiences or exercise regularly may be more likely to exercise throughout LC treatment. For these individuals, exercise may help maintain a sense of normality and control throughout treatment. Furthermore, overcoming the physical challenges of exercise can foster a sense of achievement, improving mental health. Finally, individuals who value research may also be more likely to engage.
2. Research team support
  Attendees emphasised the importance of providing educational materials to support informed decision‐making and enable wider discussions with friends and family. Materials should provide a detailed overview of the exercise programme, its potential benefits, and its potential impact on exacerbating RT side effects. Further guidance on mitigating these effects, such as increasing caloric or fluid intake, should also be provided. Information should be simple and brief as complex. lengthy documents may discourage participation. Additionally, participants should receive contact details for a designated person knowledgeable about the programme to address questions.
  Incorporating psychological support throughout the programme was also recommended. Attendees suggested allocating additional time at the first RT session to address potential anxieties. A 21‐day preparation period with pre‐treatment exercise sessions was also proposed to improve exercise confidence. Finally, celebrating programme completion was viewed as an opportunity to reinforce psychological support and signpost to ongoing exercise services, promoting long‐term behaviour change.
3. Supportive Relationships
  Attendees highlighted the value of receiving social support by inviting friends or family to exercise sessions. However, this approach should be applied cautiously, as it may increase distress for participants experiencing loneliness. Accordingly, establishing a professional rapport is essential before implementing social support strategies. For socially isolated individuals, establishing rapport with the PAC may provide an alternative source of social support, fostering exercise adherence.
4. How the study is initially presented
  Attendees emphasised that means of initial study presentation may strongly influence recruitment. Attendees recommended the treating oncologist should initially present the study, as this would signify its importance. Potential side effects and evidence limitation should also be discussed. Alternatively, specialist nurses or radiographers may be well suited to present the study due to their frequent patient contact.

## 4 Discussion

### 4.1 Summary of Main Outcomes

**Figure 3:**
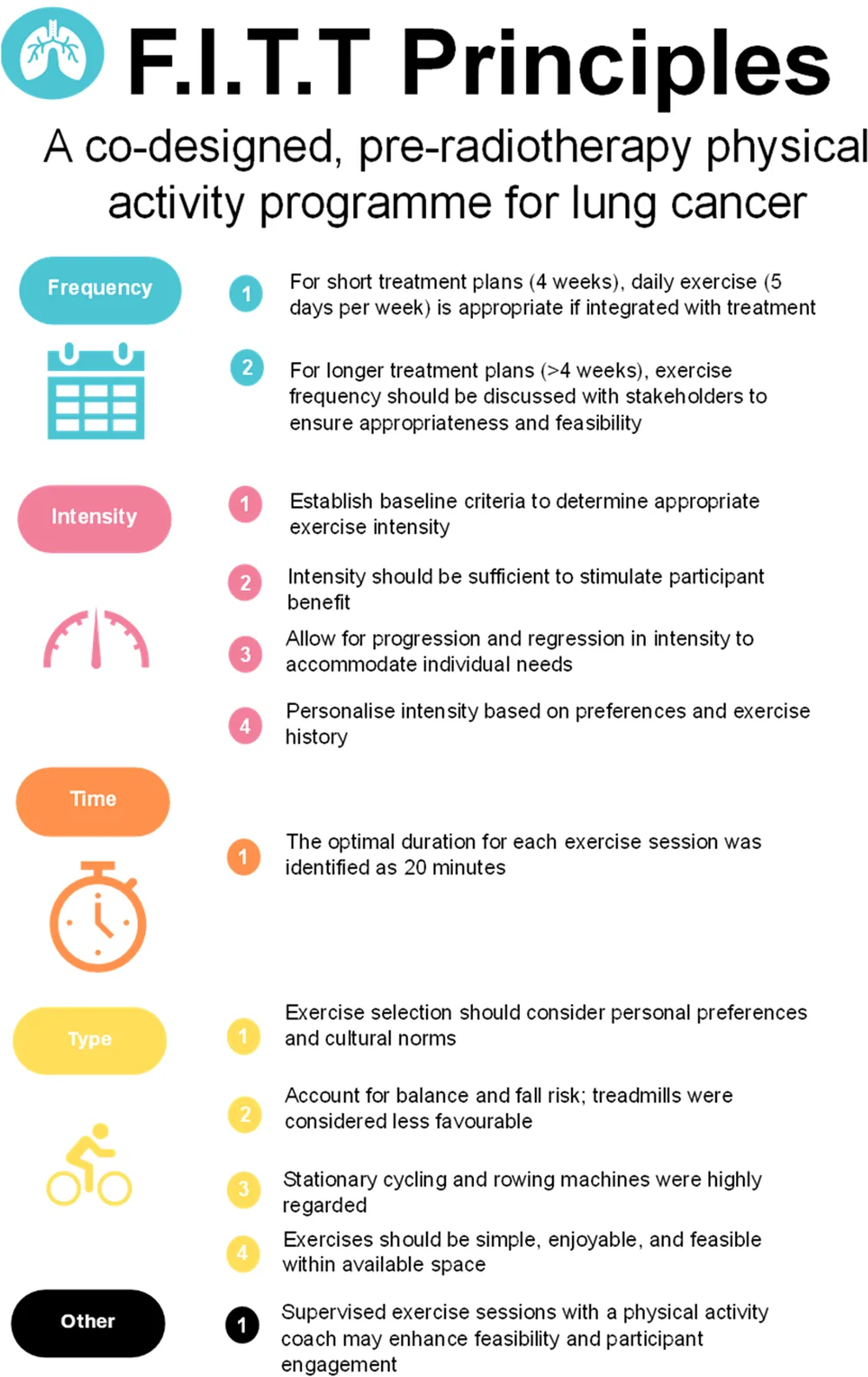
F.I.T.T principles of a co‐designed pre‐radiotherapy physical activity programme.

To our knowledge, this is the first collaborative PPIE project to co‐design a pre‐RT exercise programme for LC (Figure 3). Furthermore, through collaborative partnership, barriers to exercise participation during LC treatment were identified, and innovative solutions were developed to facilitate exercise engagement and improve the design of future exercise oncology research.

One key project outcome was the importance of creating adaptable exercise programmes, personalised to the individual. Within exercise oncology literature, fixed, ‘one‑size‑fits‑all’ programmes are increasingly recognised as a barrier, as they often fail to accommodate the diverse needs of people living with and cancer [52, 53]. To address this, researchers have incorporated autoregulation as a method of personalising exercise programmes to individual needs [54, 55]. However, autoregulation remains a novel approach. To elaborate, a recent systematic review identified 2 of 63 exercise oncology studies explicitly state autoregulation was incorporated into programme design, with few studies adapting programmes beyond baseline assessment [55].

Within this project, attendees recommended personalisation should extend beyond initial exercise prescription, with autoregulation incorporated to accommodate individuals changing needs throughout treatment. For example, prescribing multiple intensity levels may improve acceptability by enabling individuals to progress or regress intensity according to their symptoms or treatment‐related demands. However, fixed exercise prescriptions provide precision and standardisation, enabling direct comparison to evaluate efficacy [1, 3, 4]. Consequentially, autoregulation may increase heterogeneity and reduce the statistical power of efficacy trials [56, 57]. Therefore, careful implementation of autoregulation is essential to balance the benefits of personalisation with methodological rigour [56, 57].

In addition, the optimal design of exercise programme components remains an important area of investigation within exercise oncology [13]. Considering intensity, the latest evidence suggests people with cancer may gain the most benefits through exercising at moderate‐to‐high intensities, with high‐intensity interval training (HIIT) potentially offering superior outcomes [13, 58, 59, 60]. Within this project, attendees advocated for the inclusion of all exercise intensities; however, the inclusion of higher intensity exercise must be considered carefully to avoid harm [1, 4, 13]. To elaborate, people with LC often live with at least one, but often multiple co‐morbidities [61, 62]. As such, careful screening of the individual's medical history to identify co‐morbidities that may contraindicate high‐intensity exercise, such as anaemia or uncontrolled cardiovascular disease, is essential [1, 63]. This could be achieved through the use of a screening tool to triage individuals according to their risk of exercise induced adverse events [1, 4, 64], such as the Adapted National Comprehensive Cancer Network (NCCN) Triage framework [4]. Triage tools enable exercise professionals and HCP's determine the appropriate level of pre‐exercise screening, medical clearance, and supervision required according to an individual's medical history and risk profile [4, 64, 65]. Attendees reiterated this sentiment by recommending that exercise professionals adjust exercise prescriptions according to the Individual's capabilities to minimise the risk of harm. Therefore, adoption of an exercise triage tools may improve the safe delivery of a pre‐RT exercise programme.

Finally, attendees agreed that facilitating 20‑minute exercise sessions, 5 days per week, was appropriate for short duration treatment plans. This aligns with findings from a recent narrative synthesis [13], which identified short, but higher‑frequency exercise sessions were more effective for people with LC than longer programmes delivered at lower frequencies. As clinical research is limited in this area, the optimal duration of pre‐RT exercise sessions for therapeutic efficacy remains a topic of debate [21, 45]. However, outcomes from this PPIE project suggest that optimal programme design extends beyond therapeutic efficacy alone, with duration and intensity needing to be perceived by individuals as achievable and without becoming overwhelming, to sustain engagement.

### 4.2 Strengths and Limitations

A key strength of this project was the diverse stakeholder representation, including individuals with lived LC experience and their friends and family members, which fostered meaningful engagement, shared ownership, and more acceptable outcomes [66]. However, several challenges emerged. First, conducting workshops online may have inadvertently discouraged local people willing to attend in‐person, as well as limiting the intended co‐design activities [32]. Furthermore, despite extensive efforts to ensure engagement, only a small number of attendees volunteered, with only three having lived experience of LC treatment. Consequently, this limited representation may have constrained the diversity of perspectives informing discussions and resulting outcomes. These limitations reflect a broader challenge of sustaining stakeholder engagement within co‐design literature [66, 67].

To potentially address this limitation, establishing a dedicated outreach team to deliver a workshop advertisement strategy at in‐person venues and across multiple social media platforms may have facilitated broader participation and improved engagement. Alternatively, conducting the project as co‐design research with ethical approval would have permitted HCPs to approach people receiving LC treatment directly.

Additionally, scheduling conflicts prevented joint meetings between attendees and HCPs, which may have influenced outcomes. Therefore, this project did not meet the criteria to be formally classified as EBCD [31]. Finally, restricting attendee involvement to English speaking only may have limited the diversity of findings. In future co‐design work, consideration should be given to involving workshop facilitators and translators with broad language skills.

### 4.3 Implications

This project has the potential to deliver the first pre‐radiotherapy exercise programme co‐designed by people living with LC. If proven feasible, it could serve as an patient‐centred intervention for evaluation in larger clinical efficacy trials. Moreover, the co‐design methods described in this project provides a framework for future researchers seeking to integrate stakeholder input into exercise oncology research, thereby enhancing the acceptability of future studies.

## 5 Conclusion

In conclusion, by combining PPIE with co‐design methodologies, this project facilitated collaboration between people affected by LC and a PhD researcher to develop the first co‐designed pre‐radiotherapy exercise programme for individuals living with LC. This approach provides a method for future researchers who seek to incorporate PPIE into their research to enhance the acceptability and relevance of their studies. Furthermore, this project exemplifies how PPIE can meaningfully contribute to shaping the direction of future research.

## Author Contributions

**Matthew Beggs:** conceptualisation, formal analysis, methodology, visualisation, writing – original draft, writing – review and editing, project administration. **Joanne Reid:** conceptualisation, supervision, methodology, formal analysis, visualisation, writing – review and editing. **Gerard G Gerry Hanna:** conceptualisation, methodology, formal analysis, supervision, visualisation, writing – review and editing. **Abbie Edgar:** visualisation, writing – review and editing. **Seamus Cotter:** visualisation, writing – review and editing. **David Burke:** conceptualisation, visualisation, writing – review and editing. **Gillian Prue:** conceptualisation, supervision, methodology, formal analysis, visualisation, writing – review and editing.

## Funding

The authors have nothing to report.

## Conflicts of Interest

The authors declare no conflicts of interest.

## Supporting information


Supporting File 1

Supporting File 2

Supporting File 3

## Data Availability

The data generated during this study are not publicly available because participants did not consent to data sharing. The authors would be happy to share relevant anonymised excerpts from transcripts/longer quotations on reasonable request. However, all relevant data in the form of participant quotations are included within the manuscript to illustrate the key points.
