# Supplementary material for: ‘You've Given Me My Voice’—Enhancing Engagement in Exercise Oncology Research: A Patient and Public Involvement Approach to Co‑Designing a Pre‑Radiotherapy Exercise Programme With People Living With Lung Cancer
Source: Health Expect. 2026 Aug 26;29(4):e70837. doi: 10.1111/hex.70837 (PMC13509410; doi:10.1111/hex.70837)
Supplement: Supplementary file 1 [file HEX-29-e70837-s001.docx]

**Supplementary file 3: Workshop 2 Summary Sheet**

| **Need for Project:**  • Challenges with recruitment is a significant barrier for people with LC participating exercise/PA interventions.    •Currently no established pre-radiotherapy exercise program | **Overview of proposed plan and methods:**  • Prepare an opening presentation to introduce each event, revising the notes from the previous workshop and discussions with advisory group  • Roundtable discussion 1: Discuss the pre-radiotherapy exercise programme drafts, making amendments, and deciding on a final draft and which elements to prioritise if future amendments are required  • Patient Journey Mapping: complete a patient journey mapping exercise. This constitutes having a discussion of the potential barriers and facilitators which may be encountered at the following stages of a participant’s journey:  1, Diagnosis and recruitment  2, Treatment Planning  3, First day of treatment and exercise  4, Maintenance throughout radiotherapy treatment and the exercise programme  5, Treatment completion and follow-up |
| --- | --- |
| **Need for Stakeholder Involvement:**  There has only been one study research pre-radiotherapy exercise for lung cancer. This population was in a different country, and recruitment was low (44%). Therefore, there is a need for stake holder involvement to review and adapt the exercise protocol used within this study to make it more feasible for a population within Northern Ireland. | **Overview of proposed plan and methods:**  • At the end of each discussion/activity, a summary will be provided in which group consensus must be reached  • A summary of all information obtained within the workshops, which all attendees must approve before a conclusion  • In the instances of a stalemate, a group poll will be organised.  • Each activity/discussion will be allocated a time frame for engagement. The decision to 'run over time' per activity will be dependent on the researcher’s discretion |

**Citation(s):**

1. Boyd H, Mckernon S, Mullin B. Old A. Improving healthcare through the use of co-design. The New Zealand Medical Journal. 2012; 125:76-87.
2. Boyd H, McKernon S. Old A. Health Service Co-design: Working with patients to improve healthcare services guide and toolkit. 2010. https://healthcareexcellence.ca/en/resources/patient-engagement-resource/health-service-co-design-working-with-patients-to-improve-healthcare-services-guide-and-toolkit/. Accessed 14 August 2024.
