# Supplementary material for: ‘You've Given Me My Voice’—Enhancing Engagement in Exercise Oncology Research: A Patient and Public Involvement Approach to Co‑Designing a Pre‑Radiotherapy Exercise Programme With People Living With Lung Cancer
Source: Health Expect. 2026 Aug 26;29(4):e70837. doi: 10.1111/hex.70837 (PMC13509410; doi:10.1111/hex.70837)
Supplement: Supplementary file 2 [file HEX-29-e70837-s002.docx]

**Supplementary file 2: Workshop 1 Summary Sheet**

| **Need for Project:**  • Challenges with recruitment is a significant barrier for people with LC participating exercise/PA interventions.    •Currently no established pre-radiotherapy exercise program | **Overview of proposed plan and methods:**  • Prepare presentations to introduce each event  • Engage in a two-part roundtable discussion to co-design an initial draft of the pre-radiotherapy exercise program:  - Part 1 - discuss definitions and experience of exercise/physical activity, in addition to potential reasons for non-exercise participation and exercise preferences  - Part 2 - Present Egegaard et al., (2019) - Pre-radiotherapy exercise programme and discuss suitability on how elements of this programme can be adapted. This includes discussing the exercise type, levels of intensity, frequency, and duration of the programme.  Then. extend the discussion to potential methods of support that could encourage recruitment and adherence (e.g. motivational strategies). Segment responses into the following categories:  - Maintaining motivation  - Expectations of the PAC  - Supportive suggestions  - Other |
| --- | --- |
| **Need for Stakeholder Involvement:**  There has only been one study research pre-radiotherapy exercise for lung cancer. This population was in a different country, and recruitment was low (44%). Therefore, there is a need for stake holder involvement to review and adapt the exercise protocol used within this study to make it more feasible for a population within Northern Ireland. | **Overview of proposed plan and methods:**  • At the end of each discussion/activity, a summary will be provided in which group consensus must be reached  • A summary of all information obtained within the workshops, which all attendees must approve before a conclusion  • In the instances of a stalemate, a group poll will be organised.  • Each activity/discussion will be allocated a time frame for engagement. The decision to 'run over time' per activity will be dependent on the researcher’s discretion |

**Citation(s):**

1. Boyd H, Mckernon S, Mullin B. Old A. Improving healthcare through the use of co-design. The New Zealand Medical Journal. 2012; 125:76-87.
2. Boyd H, McKernon S. Old A. Health Service Co-design: Working with patients to improve healthcare services guide and toolkit. 2010. https://healthcareexcellence.ca/en/resources/patient-engagement-resource/health-service-co-design-working-with-patients-to-improve-healthcare-services-guide-and-toolkit/. Accessed 14 August 2024.
