# Supplementary material for: ‘You've Given Me My Voice’—Enhancing Engagement in Exercise Oncology Research: A Patient and Public Involvement Approach to Co‑Designing a Pre‑Radiotherapy Exercise Programme With People Living With Lung Cancer
Source: Health Expect. 2026 Aug 26;29(4):e70837. doi: 10.1111/hex.70837 (PMC13509410; doi:10.1111/hex.70837)
Supplement: Supplementary file 3 [file HEX-29-e70837-s003.docx]

**GRIPP2 Checklist**

| Section and Topic | Item | Reported on page No. |
| --- | --- | --- |
| 1: Aim | Report the aim of PPI in the study | 7 |
| 2: Methods | Provide a clear description of the methods used for PPI in the study | 7 - 15 |
| 3: Study results | Outcomes – Report the results of PPI in the study, including both positive and negative outcomes | 16 - 38 |
| 4: Discussion and conclusions | Outcomes – Comment on the extent to which PPI influenced the study overall. Describe positive and negative effects | 38 - 40 |
| 5: Reflections/critical perspective | Comment critically on the study, reflecting on the things that went well and those that did not, so others can learn form this experience | 40 |

PPI= patient and public involvement

Citation:

Staniszewska S, Brett J, Simera I, Seers K, Mockford C, Goodlad S, Altman DG, Moher D, Barber R, Denegri S, Entwistle A, Littlejohns P, Morris C, Suleman R, Thomas V, Tysall C. GRIPP2 reporting checklists: tools to improve reporting of patient and public involvement in research. BMJ (Clinical research ed.). 2017; doi:10.1136/bmj.j3453
